# Pooled Population Pharmacokinetic Analysis of Tribendimidine for the Treatment of *Opisthorchis viverrini* Infections

**DOI:** 10.1128/AAC.01391-18

**Published:** 2019-03-27

**Authors:** Isabel Meister, Piyanan Assawasuwannakit, Fiona Vanobberghen, Melissa A. Penny, Peter Odermatt, Somphou Sayasone, Jörg Huwyler, Joel Tarning, Jennifer Keiser

**Affiliations:** a Swiss Tropical and Public Health Institute, Basel, Switzerland; b University of Basel, Basel, Switzerland; c Mahidol-Oxford Tropical Medicine Research Unit, Faculty of Tropical Medicine, Mahidol University, Bangkok, Thailand; d Lao Tropical and Public Health Institute, Ministry of Health, Vientiane, Lao People’s Democratic Republic; e Department of Pharmaceutical Sciences, Division of Pharmaceutical Technology, University of Basel, Basel, Switzerland; f Centre for Tropical Medicine and Global Health, Nuffield Department of Medicine, University of Oxford, Oxford, United Kingdom

**Keywords:** liver fluke, population pharmacokinetics, tribendimidine

## Abstract

Opisthorchiasis, caused by the foodborne trematode Opisthorchis viverrini, affects more than 8 million people in Southeast Asia. In the framework of a phase 2b clinical trial conducted in Lao People’s Democratic Republic, pharmacokinetic samples were obtained from 125 adult and adolescent O. viverrini-infected patients treated with 400 mg tribendimidine following the design of a sparse sampling scheme at 20 min and 2, 7.75, 8, and 30 h after treatment using dried blood spot sampling. Pharmacokinetic data for the metabolites deacetylated amidantel (dADT) and acetylated dADT (adADT) were pooled with data from two previous ascending-dose trials and evaluated using nonlinear mixed-effects modeling. The observed pharmacokinetic data were described using a flexible transit absorption model for the active metabolite dADT, followed by one-compartment disposition models for both metabolites. Significant covariates were age, body weight, formulation, and breaking of the enteric coating on the tablets. There were significant associations between O. viverrini cure and both the dADT maximum concentration and the area under the concentration-time curve (*P* < 0.001), with younger age being associated with a higher probability of cure. Modeling and simulation of exposures in patients with different weight and age combinations showed that an oral single dose of 400 mg tribendimidine attained therapeutic success in over 90% of adult patients. Our data confirmed that tribendimidine could be a valuable novel alternative to the standard treatment, praziquantel, for the treatment of O. viverrini infections.

## Abstract (precis)

Opisthorchiasis, caused by the foodborne trematode Opisthorchis viverrini, affects more than 8 million people in Southeast Asia. In the framework of a phase 2b clinical trial conducted in Lao People’s Democratic Republic, pharmacokinetic samples were obtained from 125 adult and adolescent O. viverrini-infected patients treated with 400 mg tribendimidine following the design of a sparse sampling scheme at 20 min and 2, 7.75, 8, and 30 h after treatment using dried blood spot sampling.

## INTRODUCTION

The foodborne trematode Opisthorchis viverrini is a human liver fluke highly prevalent in Southeast Asia, especially along the lower Mekong Basin. Infection occurs via consumption of raw or undercooked cyprinid fish, mainly as part of local cultural eating habits (1–3). The disease is generally asymptomatic in its acute phase but can develop into a chronic stage that includes obstructive jaundice, gallstone formation, periductal fibrosis, and ascending cholangitis (4, 5). Moreover, O. viverrini is a major risk factor for cholangiocarcinoma, a highly invasive bile duct cancer (3).

Treatment for O. viverrini infection relies exclusively on praziquantel, but the anthelmintic agent tribendimidine, initially marketed by Chinese authorities a decade ago for the treatment of soil-transmitted helminthiasis (6), has the potential to be an alternative effective drug to combat potential resistance against praziquantel. Tribendimidine has high efficacy against O. viverrini comparable to that of praziquantel and has an excellent safety profile (7, 8). After ingestion, tribendimidine is rapidly degraded into deacetylated amidantel (dADT) and terephthalaldehyde (TPAL) without enzymatic involvement. dADT is absorbed through the intestinal wall into the blood and is partially metabolized to form acetylated dADT (adADT), while 35 to 55% is excreted unchanged in urine. TPAL is rapidly and completely metabolized to terephthalic acid (TPAC), which is also excreted in urine (9, 10). The comparative activity of tribendimidine and its metabolites, assessed *in vitro* and *in vivo* on the related Asian liver fluke (Clonorchis sinensis), reveals that the trematocidal activity is driven by dADT (11).

In the framework of a clinical development program, two phase 2a trials (68 patients) using single ascending doses of 25 to 600 mg evaluated the optimal dose of tribendimidine for the treatment of O. viverrini infections (12) in Lao People’s Democratic Republic (Lao PDR). The trials, which included patients with mainly light infection intensities, found cure and egg reduction rates above 61% and 97%, respectively, with doses of 100 mg or more. The highest cure and egg reduction rates were observed for a single oral dose of 400 mg tribendimidine (91.5 and 99.9%, respectively) or more (12). A population pharmacokinetic (PK) analysis of these data showed that both the dADT and adADT metabolites were well characterized by one-compartment disposition models with linear elimination kinetics (13). In the present study, we pooled these data with new PK data from 125 O. viverrini-infected adults treated with 400 mg tribendimidine in the framework of a phase 2b trial (7). The combined data were used to develop a pooled pharmacokinetic (PK)-pharmacodynamic (PD) model of tribendimidine metabolites in the target adult population. In addition, the PK-PD model was used to simulate exposure to the active dADT metabolite after 200- and 400-mg doses for different age and weight combinations in order to guide dosing recommendations for this novel treatment.

## RESULTS

### Participant characteristics, infections at baseline, treatment, and PK sampling.

In the phase 2b study, all 125 participants were treated as planned with a 400-mg single dose, except for 2 patients who were wrongly dosed with 200 mg and therefore included in modeling with the actual 200-mg dose received. The median age was 48 years, the median weight was 54 kg, and 54% of the participants were female (Table 1). The majority of the participants (90%, *n* = 113) had a light O. viverrini infection intensity at the baseline, with the mean egg count being 145.3 eggs per gram (EPG). From each patient, 5 PK samples were obtained, with 93% of the samples being collected within the planned windows (see Table S2 and Fig. S2 in the supplemental material). As anticipated, at the first sampling time point (20 min postdose), the concentrations of both metabolites were below the lower limit of quantification (LLOQ) in most of the samples (>85%), while overall, the concentrations were below the LLOQ in <10% of the samples at later time points.

**TABLE 1:** Summary of study design and demographics^*a*^

Characteristic	Phase 2a study			Phase 2b study (3rd trial)
	1st trial	2nd trial	1st and 2nd trials	
No. of patients	31	37		125
Dose	200 mg (*n* = 13), 400 mg (*n* = 9), 600 mg (*n* = 9)	25 mg (*n* = 9), 50 mg (*n* = 9), 100 mg (*n* = 9), 200 mg (*n* = 10)		400 mg (*n* = 123), 200 mg (*n* = 2, wrong dose)
Formulation	200-mg tablets	50-mg tablets		200-mg tablets
Median (range) age (yr)			42 (15–65)	48 (15–79)
Median (range) wt (kg)			52 (38–67)	54 (32–85)
% (no.) of female subjects			51 (35)	54 (68)
Sampling scheme			Venous blood, 0, 1, 2, 3, 4, 4.5, 5, 6, 8, 10, and 24 h; DBS, 0, 1, 3, 4.5, 6, 10 h or 0, 2, 4, 5, 8, and 24 h	DBS, 0.33, 2, 7.75, 8, and 30 h
Covariates			Age, sex, ht, wt, temp, blood pressure, parasitology, pregnancy	Age, sex, ht, wt, temp, blood pressure, parasitology, pregnancy
Other clinical data			Renal and hepatic functions (azotemia, creatinine, ASAT-GOT, ALAT-GOT)	

a DBS, dried blood spots; ASAT, aspartate aminotransferase; GOT, glutamic oxalacetic transaminase; ALAT, alanine aminotransferase.

### Pooled population PK modeling.

**FIG 1:**
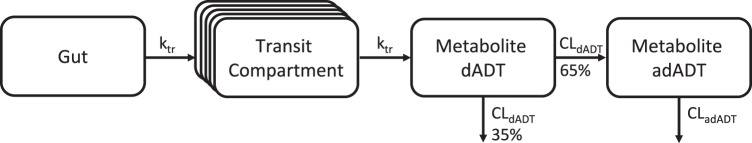
Structure of the final population PK model for tribendimidine. *k*_tr_, absorption transit rate constant; CL_dADT_, dADT clearance; CL_adADT_, adADT clearance.

One-compartment disposition models with a full Stirling approximation implementation of the transit compartment absorption model (5.27 theoretical compartments) where the absorption rate constant was assumed to equal the transit rate constant best described the observed dADT and adADT concentration-time data from the pooled studies (Fig. 1). Final population PK parameter estimates are presented in Table 2. During initial model building, there was a significant improvement in model fit when a two-compartment disposition model rather than a one-compartment disposition model was used (change in the objective function value [ΔOFV] = 102). However, this more complex two-compartment model did not provide a significant improvement in the fit of the final model compared to that of a one-compartment disposition model. The two-compartment model was also unstable, and further data would have been required to support this more complex disposition model.

**TABLE 2:** Population PK parameter estimates from the final model using pooled data from the two phase 2a trials and one phase 2b trial^*d*^

Parameter^*a*^	Population estimate (% RSE)^*b*^	95% CI^*c*^ for population estimate	% CV for BSV (% RSE)^*b*^	95% CI^*c*^ for % CV for BSV
*F* (%)	100 (fixed)		37.6 (8.20)	32.1 to 45.8
MTT (h)	3.18 (5.21)	3.05 to 3.73	54.1 (5.86)	49.9 to 66.2
No. of transit compartments	5.27 (13.4)	5.16 to 8.01	300 (6.01)	204 to 397
Metabolite dADT				
CL/*F*_dADT_ (liters/h)	15.8 (4.42)	15.1 to 17.9	19.7 (7.07)	16.6 to 22.5
*V*/*F*_dADT_ (liters)	88.8 (4.93)	84.4 to 103	25.9 (7.41)	20.1 to 28.5
Whole blood-to-plasma matrix conversion factor (%)	−14.5 (3.55)	−19.4 to −7.41		
DBS-to-plasma matrix conversion factor (%)	−13.7 (3.96)	−19.0 to −4.55		
RUV_plasma_ (% CV)	48.6 (6.30)	44.1 to 59.4		
RUV_whole blood_ (% CV)	61.2 (6.36)	52.7 to 72.8		
RUV_DBS_ (% CV)	68.8 (7.93)	54.0 to 81.7		
Metabolite adADT				
CL/*F*_adADT_ (liters/h)	65.8 (10.1)	59.6 to 87.0	116 (8.66)	105 to 172
*V*/*F*_adADT_ (liters)	15.7 (9.89)	11.5 to 18.1	30.3 (46.8)	25.9 to 54.3
Whole blood-to-plasma matrix conversion factor (%)	5.00 (1.70)	2.16 to 9.51		
DBS-to-plasma matrix conversion factor (%)	7.00 (4.15)	−1.45 to 17.7		
RUV_plasma_ (CV%)	39.4 (7.58)	31.4 to 44.3		
RUV_whole blood_ (% CV)	46.9 (6.34)	38.5 to 53.6		
RUV_DBS_ (% CV)	46.7 (10.8)	37.1 to 63.7		
Covariate effects				
Age on CL/*F*_dADT_ (%)	−1.19 (9.14)	−1.35 to −0.88		
Formulation on MTT (%)	42.9 (29.2)	16.0 to 61.4		
Split 50-mg tablets on MTT (%)	−79.4 (12.1)	−100 to −60.2		

a Population parameter estimates are presented for a typical patient at 45 years of age weighing 52 kg and receiving the 200-mg formulation as whole tablets and with drug concentrations measured in plasma.

b Computed population mean values are from NONMEM. Between-subject variability (BSV) is calculated as $\sqrt{\text{exp}\left(\text{variance}\right)-1}$. Relative standard errors (RSEs) are calculated as 100(standard deviation/mean value).

c Based on nonparametric bootstrap diagnostics of the final PK model (*n* = 1,000). The 95% confidence intervals (CI) are displayed as the 2.5th to the 97.5th percentile of bootstrap estimates.

d The two phase 2a trials included 68 patients and have been described previously (16), and the phase 2b trial included 125 patients. dADT, deacetylated amidantel; adADT, acetylated dADT; *F*, relative bioavailability; MTT, mean absorption transit time; CL/*F*, apparent elimination clearance; *V*/*F*, apparent volume of distribution; CV, coefficient of variation; DBS, dried blood spots; RUV_plasma_, residual unexplained variability for plasma; RUV_whole blood_, residual unexplained variability for whole blood; RUV_DBS_, residual unexplained variability for DBS; Age on CL/*F*_dADT_, linear covariate relationship between age and CL/*F*_dADT_ centered on the median age of 45 years; Formulation on MTT, the formulation effect on MTT (200 mg or 50 mg); Split 50-mg tablets on MTT, the effect of breaking the administered tablets (i.e., a 50-mg tablet broken in half) on MTT.

A systematic conversion factor between plasma and whole blood concentrations improved the model fit significantly (ΔOFV = 73), resulting in 13.7 to 14.5% lower and 5 to 7% higher drug concentrations in plasma than in blood for dADT and adADT, respectively. The difference between drug concentrations measured in whole blood and dried blood spots (DBS) was not statistically significant.

Different drug formulations of 50 mg and 200 mg had a significant impact on the absorption transit rate constant (ΔOFV = 56.7), resulting in a 42.9% slower mean absorption transit time for the 200-mg formulation than for the 50-mg formulation. In addition, breaking the tablet and thereby destroying the enteric coating (as was done for some of the patients in the phase 2a trials) had a significant impact on the absorption transit rate constant (ΔOFV = 79), resulting in a 79.4% faster mean absorption transit time for broken tablets than for whole 50-mg tablets. Age was found to be a significant linear covariate on dADT elimination clearance (ΔOFV = 30.8), resulting in a modest 1.19% decreased clearance per year increase in age.

**FIG 2:**
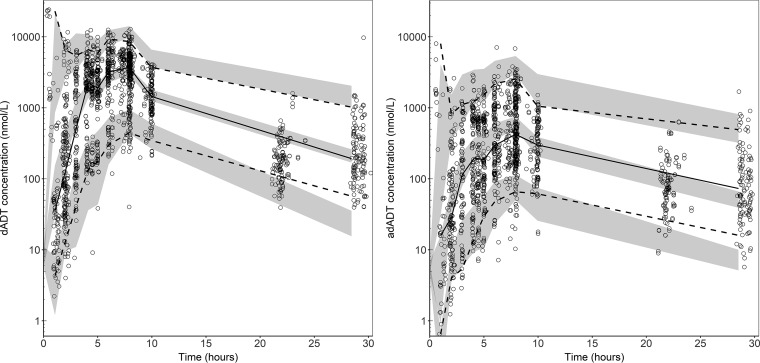
Prediction-corrected visual predictive checks for dADT (left) and adADT (right) for the final population PK model. Black circles represent observed data. Dashed lines represent the 5th and 95th percentiles of observed data. Solid lines represent the 50th percentiles of observed data. Grey bands are the 95% confidence intervals for the corresponding simulated 5th, 50th, and 95th percentiles.

Overall goodness-of-fit diagnostics showed a reasonable model fit without any obvious model misspecification (Fig. S5 and S6). However, the final model resulted in relatively high eta shrinkages associated with certain variability parameters (i.e., eta shrinkages were 31% for apparent elimination clearance for dADT [CL/*F*_dADT_], 38% for apparent volume of distribution for dADT [*V*/*F*_dADT_], 31% for relative bioavailability [*F*], and 66% for apparent volume of distribution for adADT [*V*/*F*_adADT_]), and the individual model fits should be interpreted with caution. The prediction-corrected visual predictive check (pcVPC) for dADT showed an adequate predictive performance with some model misspecification of the median percentile in the absorption phase and of the 5th percentile in the disposition phase (Fig. 2, left). The pcVPC for the inactive metabolite, adADT, presented overpredicted peak concentrations of the median percentile (Fig. 2, right). The pcVPCs for the fraction of the data below the LLOQ for dADT (Fig. S3) and adADT (Fig. S4) showed some model misspecification during the absorption phase, particularly in the DBS matrix. However, the misspecification occurred before the peak concentrations. Therefore, caution should be applied if the model is used for extrapolation during early absorption.

The PK parameter estimates are summarized in Table 3. As expected, dADT exposure increased proportionally with dose, ranging from a maximum concentration (*C*_max_) of 61.4 ng/ml and an area under the concentration-time curve (AUC) of 509 ng·h/ml with the 25-mg dose to a *C*_max_ of 1,271 ng/ml and an AUC of 12,230 ng·h/ml for the 600-mg dose. Overall adADT exposure was also proportional to the dose for all doses except the 200-mg dose, where the *C*_max_ and AUC achieved with the 200-mg dose appeared to be lower than those achieved with the 100-mg dose.

**TABLE 3:** Secondary PK parameter estimates^*a*^

Dose (mg)	No. of subjects	*C*_max_ (ng/ml)	*T*_max_ (h)	Half-life (h)	AUC (ng·h/ml)
dADT					
25	9	61.4 (55.5–71.7)	1.73 (1.22–2.03)	4.03 (3.67–5.03)	509 (418–525)
50	9	173 (160–228)	4.86 (3.87–8.15)	3.34 (2.96–3.42)	1,152 (1,042–1,489)
100	9	315 (279–366)	3.73 (2.56–5.55)	3.68 (3.46–4.07)	2,310 (1,966–2,433)
200	25	509 (411–722)	6.59 (4.57–9.40)	4.21 (3.65–4.92)	5,382 (4,280–6,477)
400	132	863 (594–1,102)	8.56 (6.84–11.1)	4.22 (3.63–5.15)	9,889 (7,871–12,758)
600	9	1,271 (626–1,254)	10.0 (3.95–10.9)	4.56 (3.95–5.16)	12,230 (10,736–17,794)
adADT					
25	9	23.2 (4.30–32.0)	2.64 (2.01–2.84)	4.03 (3.67–5.03)	160 (40.3–224)
50	9	67.0 (13.6–68.7)	5.34 (4.40–8.22)	3.34 (2.96–3.42)	372 (114–518)
100	9	111 (95.3–123)	4.98 (3.16–5.74)	3.68 (3.46–4.07)	771 (639–987)
200	25	60.8 (32.0–227)	6.82 (4.80–9.47)	4.21 (3.65–4.92)	614 (365–2,165)
400	132	107 (57.8–264)	8.79 (7.02–11.8)	4.41 (3.72–5.21)	1,479 (740–3,660)
600	9	253 (235–361)	10.2 (4.28–11.1)	4.97 (3.95–5.17)	3,748 (2,678–5,050)

a *C*_max_, maximum concentration; *T*_max_, time to reach maximum concentration; AUC, area under the concentration-time curve (0 to 72 h). All values are presented as the median (range).

### PK-PD analysis and exposure simulations.

**FIG 3:**
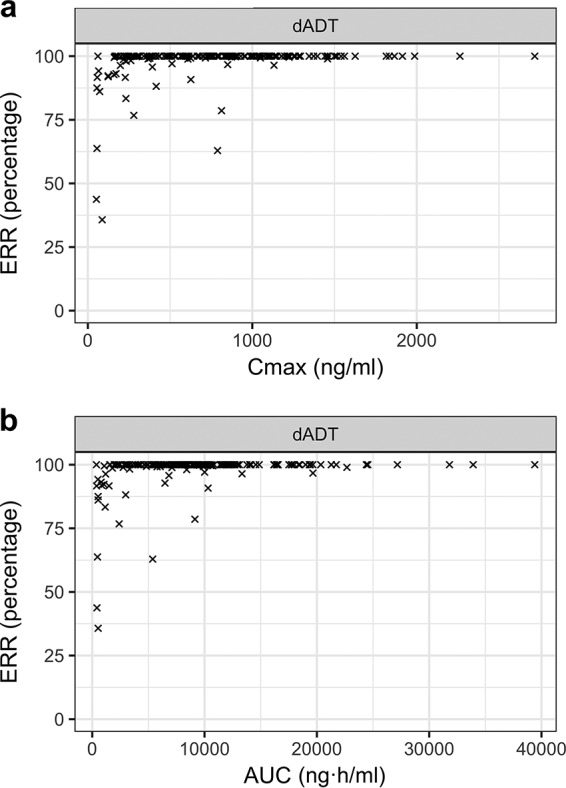
Egg reduction rate (ERR) in O. viverrini among participants (*n* = 190) from the 3 trials plotted by the exposure parameters *C*_max_ (a) and AUC (b).

In the present phase 2b study, 1 patient was lost at follow-up, failing to present for the final PD assessment at 21 days following treatment. PK participants in the trial (*n* = 124) had an average O. viverrini egg reduction rate (ERR) of >99%, and 93% were cured of O. viverrini infection. Across all three trials, 151/191 (79%) participants were cured, and visually, there was a clear exposure-response relationship for O. viverrini infections, with higher *C*_max_ or AUC associated with higher ERRs (Fig. 3a and b). After adjustment for confounders, there were strong associations between O. viverrini cure and both the dADT *C*_max_ and the dADT AUC (*P* < 0.001; Table S3). Of note, younger age was associated with a higher probability of cure, after adjustment for exposure.

**FIG 4:**
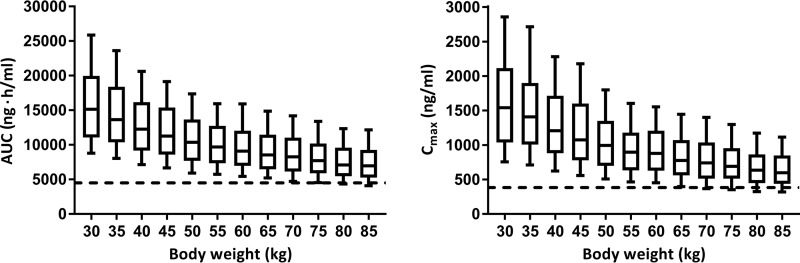
Simulations (*n* = 1,000) of the exposure, namely, AUC (left) and *C*_max_ (right), stratified by body weight after administration of a single oral dose of 400 mg tribendimidine. The dashed horizontal lines indicate the AUC criterion (left) and the *C*_max_ criterion (right) associated with the 90% probability of achieving a cure. The AUC criterion is 4,520 ng·h/ml, and the *C*_max_ criterion is 384 ng/ml. The box and whiskers represent 25th to 75th and 10th to 90th percentiles, respectively.

In the univariable regression models of O. viverrini cure rates against exposure, the 90% probability of achieving a cure was estimated at a *C*_max_ of 384 ng/ml and an AUC of 4.520 ng·h/ml. Population-based simulations of the *C*_max_ and AUC of dADT, stratified by body weight (30 to 85 kg), from the final PK model suggested that 400-mg flat dosing achieved the *C*_max_ criterion of 384 ng/ml and the AUC criterion of 4,520 ng·h/ml for all body weights (Fig. 4). A high variability in exposure was observed for all weight groups.

## DISCUSSION

Our pooled PK-PD study of tribendimidine in O. viverrini-infected adults and adolescents in Lao PDR confirmed that a single dose of 400 mg achieved adequate exposure and high efficacy (cure rates, >90%). This study adds to the evidence that tribendimidine is a promising alternative treatment against opisthorchiasis, which, when untreated, can result in potentially serious adverse health outcomes, including cholangiocarcinoma (3).

The data obtained generated robust parameter estimates with reasonable precision, therefore supporting the appropriateness of the current sampling design. The PK properties of the main metabolites of tribendimidine, dADT and adADT, were best described by a flexible transit absorption model followed by a one-compartment disposition model with first-order elimination. Although data below the LLOQ were handled by using Beal’s M3 method (14), some model misspecification in the absorption phase remained for dADT. However, despite this potential misspecification, the peak concentrations were adequately predicted. Overpredicted peak concentrations were seen with adADT, but given adADT’s minor role in the drug’s activity, this should not be of any major concern in regard to therapeutic efficacy or dosing assessments. A two-compartment disposition model for dADT initially resulted in an improved model fit. However, when this initial structural model was rechallenged using the final model, a two-compartment disposition model was not statistically superior to a one-compartment disposition model. The two-compartment disposition model was also unstable, and small perturbations of initial estimates resulted in different parameter estimates, most likely reflecting the fact that not enough data were collected to support a more complex structural disposition model.

Findings from the pooled analysis with respect to the model structure and covariate effects corresponded to previously published PK modeling results for tribendimidine (13). Clearance and volume parameters were scaled allometrically with body weight. After adjustment for differences in body size, the clearance of dADT decreased approximately 10% per 10-year increase in age, which might be due to declining renal function with age (15). The mean absorption transit time was affected by the formulation and tablet splitting. The pooled analysis combined data from a range of single-dose treatment levels with two different enteric coating formulations. The 200-mg tablets were used in the present phase 2b study, but drug administration in the phase 2a studies included a novel 50-mg formulation for some patients. The 200-mg formulation resulted in slower absorption than those achieved with the 50-mg formulation. This was previously explained by the reported *in vitro* physicochemical properties of the 200-mg formulation making it more likely to float in the stomach, resulting in delayed absorption (16). Breaking of the tablets resulted in faster absorption. This is expected, since the destruction of the enteric coating is likely to cause an immediate release of the drug (16). In contrast to the findings of Vanobberghen and colleagues (13), we found that the addition of a conversion factor between plasma and blood concentrations for dADT and adADT improved the model fit significantly.

We observed an exposure twice as high in O. viverrini-infected adolescents and adults as in healthy Chinese volunteers (AUC, 9,889 ng·h/ml versus 4,769 ng·h/ml [17] or 4,450 ng·h/ml [9], respectively). In African children, an even higher total area under the concentration-time curve from 0 h to infinity (AUC_0–inf_) of 12,530 ng·h/ml was observed (18). In all the studies, the same commercially available 200-mg tablets were used; however, the studies differed with regard to the food intake or the fasting of the participants. Whether the much higher exposure observed in O. viverrini-infected participants than in healthy volunteers was achieved due to food intake or to disease physiopathology is an important question which should be addressed in future research. With the flotation characteristic of currently marketed tribendimidine tablets, the question of treating fasted or nonfasted patients could play an important role when assessing the between-patient variability in dADT exposure.

In summary, the pooled population PK model presented here contributes to a better understanding of the modalities of exposure to the main metabolites of tribendimidine, dADT and adADT, in adult and adolescent patients. Simulations using the final PK-PD model showed that a single oral 400-mg dose of tribendimidine can uniformly achieve a drug exposure associated with O. viverrini cure rates above 90%. The modeling conducted here confirmed that the formulation had a significant impact on the absorption of tribendimidine. This pooled study supports the development of tribendimidine as a novel opisthorchicidal drug, but given the demonstrated variability in drug absorption, there is a need to consider refinements to the current formulation. As noted above, tribendimidine presents significant advantages over standard treatment with praziquantel: a single and body weight-independent dose regimen that also causes fewer side effects (7). In conclusion, tribendimidine is a promising alternative for the treatment and control of opisthorchiasis.

## MATERIALS AND METHODS

### Study design and ethical considerations.

The two phase 2a trials involving 68 patients treated with 50 to 600 mg tribendimidine were described previously (16). A noninferiority randomized controlled phase 2b trial comparing tribendimidine (400 mg) and praziquantel (75 mg/kg) for the treatment of O. viverrini infections was carried out in 607 adults and adolescents between February and April 2014 in the district of Champasack in Lao PDR (7). Among the 295 patients from the tribendimidine treatment arm, a subset of 125 adults, enrolled consecutively from those presenting in the morning and giving consent, underwent PK sampling. Ethical clearance was given from the Ethics Committee of Northwestern and Central Switzerland (EKNZ reference no. 375/11), the National Ethics Committee in Laos (009/NECHR), and the Liverpool School of Tropical Medicine (reference no. 12.02RS). The trial is registered at the ISRCTN registry (ISRCTN96948551). Written, informed consent was obtained from all the patients before enrollment into the study.

### Patients, treatment, and study procedures.

Prior to the beginning of the phase 2b trial, screening for positive patients was performed with duplicate Kato-Katz thick smears (19) on two stool samples collected within 5 consecutive days. Participants underwent a full medical checkup, including measurement of weight, height, blood pressure, and axillary temperature and pregnancy assessment for female participants. The rural setting of the trials did not allow the assessment of further biochemical and clinical parameters (7). Eligible participants revealed no major systemic or chronic illness or psychiatric or neurological disorders and were not pregnant. The participants were treated with a single oral dose of 400 mg tribendimidine using enteric-coated tablets of 200 mg (Shandong Xinghua Pharmaceutical Corporation, Zibo, China) after a standard meal and were provided with water *ad libitum*. Adverse effects were monitored over 24 h after drug administration. After 21 days posttreatment, the patients underwent a second parasitological examination to assess treatment efficacy using, again, 2 stool samples. Values of the number of eggs per gram (EPG) were determined by adding the egg counts of the 4 slides followed by multiplication by a factor of 6 (20). Cure of the participants was defined as the percentage of patients that were negative for O. viverrini infection (absence of eggs) at follow-up. Egg reduction rates (ERR) were calculated using the following formula: ERR = [1 − (geometric mean EPG at follow-up/geometric mean EPG at baseline)]·100.

### PK sampling and analysis.

Dense PK sampling was performed in the phase 2a studies, collecting venous blood samples at 0, 1, 2, 3, 4, 4.5, 5, 6, 8, 10, and 24 h postdose for both blood and plasma concentration measurements. In addition, DBS samples were collected at 0, 1, 3, 4.5, 6, and 10 h for half of the patients, while the other half was sampled at 0, 2, 4, 5, 8, and 24 h (16). A previous preliminary analysis of these data was used to optimize a sparse PK sampling schedule in the phase 2b study (see the files in the supplemental material). The sampling scheme in the phase 2b study consisted of the use of five DBS samples taken from each patient at 0.32, 2.00, 7.75, 8.00, and 30.0 h after treatment within the sampling windows of 0.27 to 0.85, 1.18 to 2.57, 6.28 to 7.85, 7.88 to 8.00, and 28.6 to 30.0 h, respectively (Table 1, Table S1, and Fig. S1). Four blood drops were collected from each participant’s fingertip using lithium heparin-coated capillaries (volume = 40 μl; Alere 238 Cholestech LDX) and deposited onto filter paper DBS cards (DMPK-C cards; Whatman, GE Healthcare Life Sciences, Cardiff, UK). Samples were dried for 3 h before being transferred and stored in plastic bags with silica desiccants at 4°C. Tribendimidine, dADT, and adADT in the DBS samples were analyzed following a previously published liquid chromatography-tandem mass spectrometry (LC-MS/MS) method (21). Briefly, the LC-MS/MS system was composed of a quaternary high-performance liquid chromatograph (Shimadzu, Kyoto, Japan) coupled to an API 3000 tandem mass spectrometer (AB Sciex, MA, USA). The analytical range for both metabolites was 1 to 2,000 ng/ml. The calibration curves were prepared with blood with a hematocrit value of 35%, and quality control (QC) samples were assayed at 4 different concentrations in 6 replicates each using blood with hematocrit values ranging from 25% to 50%. Acceptance criteria for each analytical run were a mean accuracy of 85 to 115% (80 to 120% at the lower limit of quantification) for the calibration curve and for 4 out of 6 QC samples at each concentration.

### Pooled population PK modeling.

The population PK model for tribendimidine was developed using pooled data from the 3 studies (two phase 2a trials, *n* = 68 patients; one phase 2 b trial, *n* = 125 patients) (7, 12). All trial-specific details relevant to the PK analysis, including the tablet formulations and doses, are summarized in Table 1. Population PK properties for dADT and adADT were characterized using nonlinear mixed-effects modeling in the software NONMEM (version 7.3; ICON Development Solutions, Ellicott City, MD, USA). The concentrations of dADT and adADT were transformed into their natural logarithms and modeled sequentially using the population PK parameters and data (PPP&D) method (22). To account for values below the LLOQ, occurring predominantly in the absorption phase (13% for dADT and 21% for adADT in all matrices), Beal’s M3 method was used (14).

**(i) Structural and stochastic model development.** One-, two-, and three-compartment structural models were explored to characterize the PK properties of dADT and adADT. The percentage of dADT cleared renally was fixed at 35%, based on a previously reported value (9), and the remaining 65% was assumed to be metabolized to adADT. Zero-order and first-order absorption with and without a lag time was explored for the absorption process. Implementation of a transit compartment absorption model, using the Stirling approximation (23), as well as adding transit compartments in a stepwise manner, was also evaluated. Relative bioavailability was fixed to unity, but between-subject variability (BSV) was allowed. Population-based conversion factors were assessed for each of the compounds to compensate for systematic differences between concentration measurements in different sampling matrices (i.e., venous plasma and whole blood as well as DBS) (24). A hypothetical intermediate transfer compartment from dADT to adADT was evaluated to describe the formation of rate-limited elimination previously reported for adADT (13). Additive and combined error models on log-transformed data were evaluated to describe the residual unexplained variability. BSV was implemented using exponential models.

**(ii) Covariate analysis.** Potential covariates were assessed based on statistical significance, biological plausibility, and prior knowledge of factors influencing the PK properties of dADT and/or adADT (13). Total body weight was implemented *a priori* as an allometric function, centered on the median body weight, on clearance and volume parameters simultaneously using a fixed exponent of 0.75 for clearance and 1 for volume (25). Other covariates tested included age, sex, formulation, and the effect of breaking the administered tablets (i.e., a 50-mg tablet broken in half in the phase 2a studies). Creatinine clearance data were not available for all patients and could not be considered in this analysis. Covariates were evaluated using a forward selection (*P* < 0.05) and a backward elimination (*P* < 0.001) approach. Linear, exponential, and power parameter-covariate relationships, centered on their median values for the population, were tried.

**(iii) Model discrimination and evaluation.** Model discrimination was based on the likelihood ratio test for nested models. A decrease in the objective function value (OFV) of 3.84 was considered statistically significant (*P* < 0.05) when comparing hierarchical models with a 1-degree-of-freedom difference. Models were evaluated using goodness-of-fit and simulation-based diagnostics (prediction-corrected visual predictive checks [pcVPCs]; *n* = 1,000). In addition, the final model was evaluated using nonparametric bootstrap diagnostics (*n* = 1,000), stratified by study, to obtain relative standard errors (RSEs; in percent) and 95% confidence intervals (CI) of the parameter estimates.

### PK-PD analysis.

The PK parameters evaluated for each metabolite included the maximum concentration (*C*_max_), time to maximum concentration (*T*_max_), elimination half-life (*t*_1/2_), and total area under the concentration-time curve from 0 h to infinity (AUC_0–inf_), which were derived from the final pooled population PK model for all individuals in the three studies. To assess the relationship between treatment outcome and drug exposure, cure of the O. viverrini infection as a function of the dADT *C*_max_ or AUC was modeled separately using multivariable logistic regressions with sex, age, and body mass index as potential confounders.

Univariable logistic regressions of cure of O. viverrini infection against the dADT *C*_max_ or AUC were also performed to obtain a therapeutic criterion. The therapeutic criterion was defined as a *C*_max_ or AUC value associated with a 90% probability of curing the infection (*C*_max_ criterion or AUC criterion) (R software, version 3.3.1).

### Simulations of drug exposure stratified for weight.

A single oral dose of 400 mg of tribendimidine has been demonstrated to produce a cure rate of 92.6% in adults and adolescents aged ≥15 years (7). Thus, the final PK model was used to simulate a single oral dose of 400 mg in patients with a typical age of 45 years and a body weight range of 30 to 85 kg at increments of 5 kg. A total of 1,000 virtual patients were simulated per patient characteristic. The simulation was performed using the 200-mg formulation since it is the most commonly used formulation. The simulation was performed using the 200-mg formulation since it is the commonly used formulation. Simulated dADT *C*_max_ and AUC stratified by body weight were plotted to assess the probability of achieving therapeutic success at different body weights. Successful treatment was defined as *C*_max_ or AUC values being above the *C*_max_ criterion or AUC criterion.

## Supplementary Material

Supplemental file 1
