# Supplementary material for: Pooled Population Pharmacokinetic Analysis of Tribendimidine for the Treatment of Opisthorchis viverrini Infections
Source: Antimicrob Agents Chemother. 2019 Mar 27;63(4):e01391-18. doi: 10.1128/AAC.01391-18 (PMC6437521; doi:10.1128/AAC.01391-18)
Supplement: Supplemental file 1 [file AAC.01391-18-s0001.pdf]

SUPPLEMENTARY MATERIAL

Optimal sample design

Methods

The determination of an optimal design for our study was based on PK data from a previous phase 2a study (Duthaler *et al.*, 2016). The data included 309 dried blood spot samples taken over 24 h from 31 patients, from which dADT and adADT concentrations were determined. Firstly, we determined a structural PK model using methods previously described, although the model used differed slightly from that previously published (Duthaler *et al.*, 2016). Secondly, we defined a range of sampling designs meeting our logistical constraints, which were a maximum of 600 samples, a minimum of 15 min between two consecutive samples, and sampling windows between 0-8 and 24-30 h post-dose. The sampling designs varied in the number of patients and number of samples per patient, with the goal of minimising the number of samples per patient while keeping the number of patients to recruit low. All scenarios included at least one time point before 1 h, a sample fixed at 2 h was incorporated to offer protection against measures before 2 h being below the limit of quantification. The software WinPOPT version 1.2 (Mentré *et al.*, 2007) applied to the structural model was used to choose the optimal design, by maximising the determinant of the Fisher matrix and hence minimising the standard errors (SEs) (Savic *et al.*, 2007).

The upper acceptability limit for the predicted SEs was set at 25% (Savic *et al.*, 2007). During sampling scheme comparisons, the exchange algorithm was used to determine the sampling times; for the final model, simulated annealing was used. A window around the sampling times was allowed for logistical reasons allowing a maximum of 20% increase in the SEs. In practice, variation in sampling times within the specified sampling windows may be preferred to safeguard against an incorrectly specified structural model (Mentré *et al.*, 2007). The sampling design was optimised for dADT, the most active metabolite (Xiao *et al.*, 2009).

Results

We used a one-compartment disposition model for each metabolite, with an absorption model with four transit-compartments. Inter-individual variability (IIV) was incorporated on the adADT clearance (CL/F) and mean transit time parameters only. The final parameters were dADT CL/F 16.4 L/h, dADT volume of distribution (V_C/F) 102 L, adADT CL/F 64.3 L/h (IIV=0.578), adADT V_C/F 20.4 L, mean transit time 6.55 hours (IIV = 0.269) and residual errors σ^2 0.707 for dADT and 0.265 for adADT.

A sampling scheme with 100 patients and 6 samples per patient at naively-chosen times resulted in a large SE for dADT V_C/F and low determinant of the Fisher matrix (Table S1). Optimising the sampling times for a similar scheme in 100 patients with 6 samples resulted in relative SEs <25% and a much larger Fisher determinant. Reducing the number of patients to 80 and increasing the number of samples to 7 per patient yielded relative SEs <25% but a lower Fisher determinant. Instead increasing the number of patients to 120 and reducing the number of samples to 5 per patient yielded relative SEs <25% and a larger Fisher determinant. This scheme was also considered preferable in terms of balancing the number of patients versus samples per patient. In addition, we considered schemes with two groups of patients with different sampling times but none met the criterion of SEs <25% (data not shown). For the final scheme with 5 samples in each of 120 patients, the optimised sampling times (windows) were 0.32 (0.27-0.85), 2.00 (1.18-2.57), 7.75 (6.28-7.85), 8.00 (7.88-8.00) and 30.0 h (28.6-30.0 h) (Figure S1). This set of sampling times included time points around the peak of the concentration by time curve, and around 24 hours, as anticipated. The windows had an estimated efficiency of 96%, i.e. a predicted increase in SEs of only 4% compared to the sampling scheme without windows.

Table S1 Predicted performance for a selection of sampling scenarios

Total N samples (n patients: n samples/patient)	Sampling times	Relative SE[a] (%)					Determinant of Fisher matrix
		CL/F	V_C/F	MTT (h)	IIV for MTT	dADT residual error	
600 (100:6)[b]	1, 2, 4, 6, 8, 24	15.4	30.6	10.8	20.6	3.2	172
600 (100:6)	0.327, 0.601, 2, 7.73, 8, 28.7	15.5	25.0	8.5	18.9	3.2	551
560 (80:7)	As 100:6 above, plus 6.94	15.0	24.0	9.1	20.9	3.2	304
600 (120:5)[c]	0.313, 2, 7.75, 8, 30	14.5	22.8	8.3	19.6	3.2	848

SE, standard error; CL/F, dADT clearance; V_C/F, dADT volume of distribution; MTT, mean transit time; IIV, inter-individual variability.

[a]SEs above the *a priori* limit of 25% are highlighted in dark grey. Higher values of the determinant of the Fisher matrix were desired. Restrictions were: maximum 600 samples, minimum 15 minutes between samples, and samples taken between 0-8 and 24-30 hours. In addition, we fixed a sample at 2 hours. Optimisation performed by the exchange algorithm except for the final sampling design (last row) where simulated annealing was used.

[b]Naively-chosen sampling times (ie not optimised), to illustrate the predicted high SE and low determinant of the Fisher matrix.

[c]Chosen sampling scheme.

Table S2 Sampling summary

Target as indicated by optimal design		Actual sampling			
Sampling time (h)	Window (h)	Mean time (h) [range]	N (%) samples within the window	N (%) dADT samples below the LLOQ	N (%) adADT samples below the LLOQ
0.3	0.27,0.85	0.52 [0.25,0.85]	122 (98%)	108 (86%)	113 (90%)
2	1.18,2.57	1.82 [1.18,2.50]	125 (100%)	9 (7%)	52 (42%)
7.8	6.28,7.85	6.97 [6.10,7.93]	116 (93%)	0 (0%)	0 (0%)
8	7.88,8	7.93 [7.67,8.92]	99 (79%)	0 (0%)	0 (0%)
30	28.6,30	29.06 [28.35,30.33]	118 (94%)	4 (3%)	12 (10%)

N=125 for each time point (total of 625 samples)

Table S3 Regression analyses for cure rates

	Univariable models		Multivariable model with dADT Cmax		Multivariable model with dADT AUC	
	Odds ratio (95% CI)	P value	Odds ratio (95% CI)	P value	Odds ratio (95% CI)	P value
Sex, female	1.46 (0.73-2.94)	0.29	1.21 (0.55-2.66)	0.64	1.26 (0.57-2.80)	0.57
Age, per 10 years	0.92 (0.73-1.15)	0.45	0.71 (0.53-0.95)	0.02	0.61 (0.44-0.84)	0.002
BMI, per 5 kg/m^2	1.13 (0.69-1.85)	0.64	1.60 (0.88-2.89)	0.12	1.27 (0.70-2.30)	0.43
dADT C_{max}, ng/ml		<0.001		<0.001	-	-
<4000	1 (reference)		1 (reference)			
4000-<7000	3.86 (1.37-10.9)		4.24 (1.45-12.4)			
7000-<10,000	5.79 (2.11-15.9)		6.85 (2.39-19.6)			
≥10,000	10.8 (3.40-34.0)		20.1 (5.35-75.7)			
dADT AUC ng/ml/h		<0.001	-	-		<0.001
<5000	1 (reference)				1 (reference)	
5000-<9000	5.89 (2.20-15.8)				7.16 (2.45-20.9)	
9000-<12,000	4.13 (1.58-10.8)				6.17 (2.12-18.0)	
≥12,000	14.1 (3.82-51.7)				43.6 (8.87-214)	

CI = confidence interval, C_{max} = maximal concentration, AUC = area under the curve, BMI = body-mass index. Non-linearity was assessed for age, weight, and BMI but none detected. Categories for C_{max} and AUC chosen based on frequency distribution. Results based on 191 individuals, of whom 151 (79%) were cured.

Figure S1 Predicted dADT concentration over time, with optimised sampling scheme and sample windows.

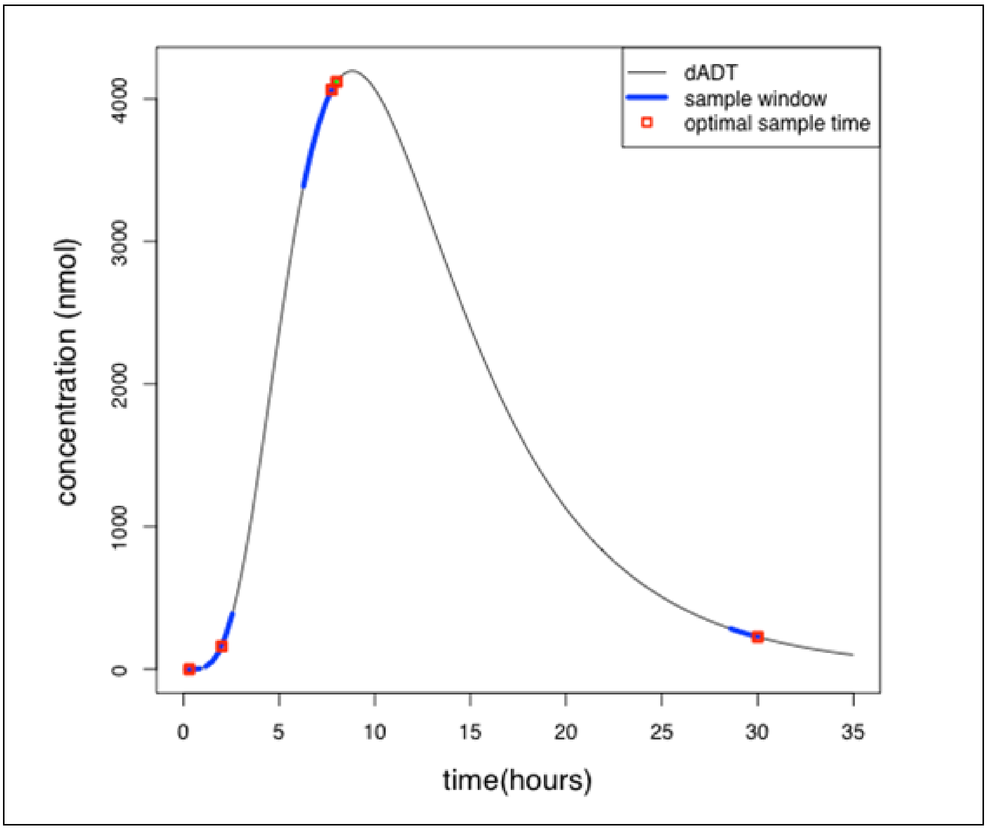

Figure S2 PK profiles of dADT and adADT in the patients from the phase 2b trial (400 mg dose, n=123). Median concentrations and their 95% confidence intervals calculated for each nominal time point are represented by white squares and shaded area, respectively.

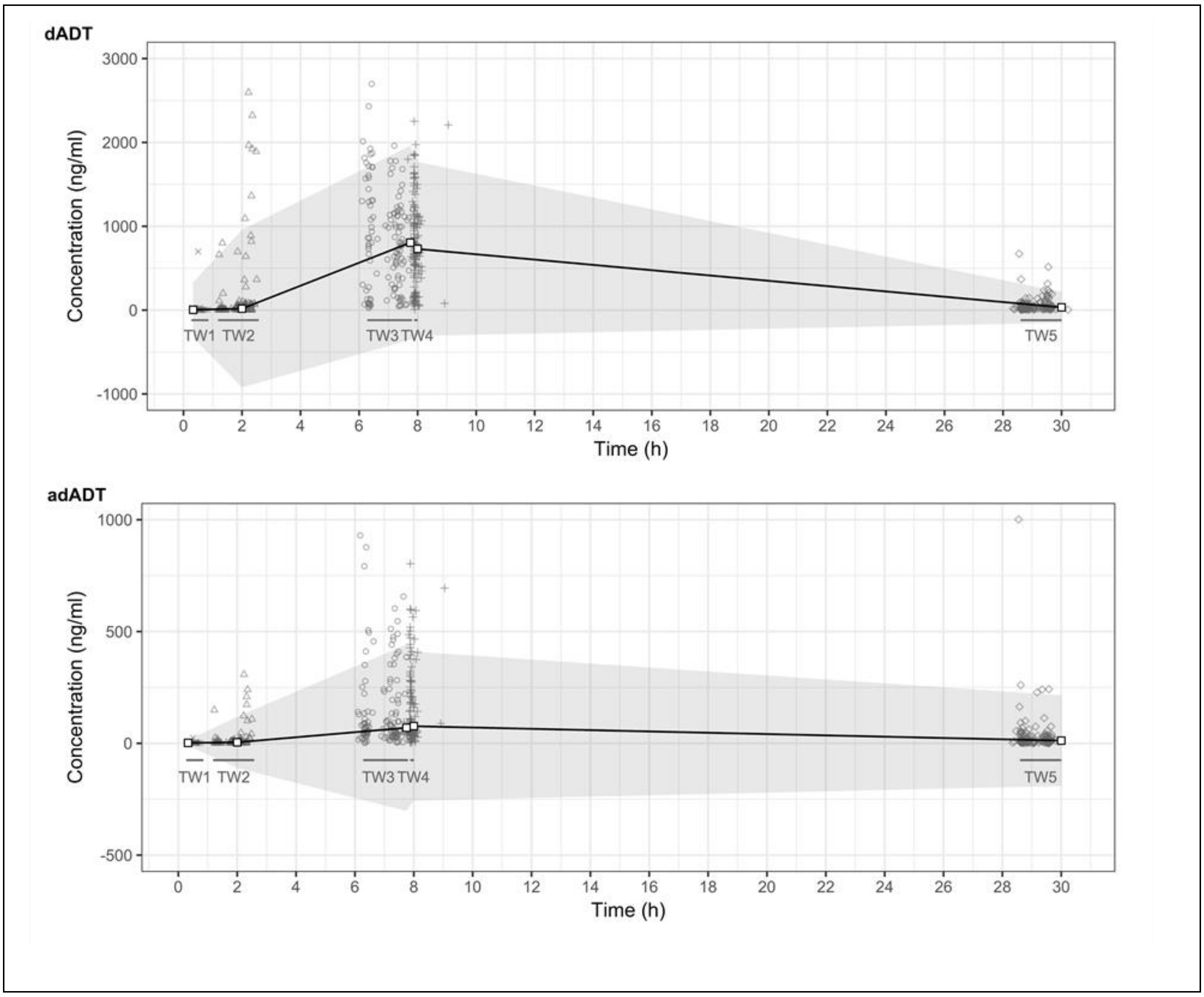

The grey horizontal lines represent the five planned sampling time windows (TW1 to 5). The grey shapes correspond to the individual concentrations (each shape being assigned to a different nominal time point), arranged on the x-axis by their real sampling time (data below LLOQ are omitted as well as the data of two patients wrongly dosed with 200mg).

Figure S3 The fraction of observed data below the LLOQ (open circles) overlaid with the 95% prediction interval of the fraction of simulated data below the LLOQ (shaded area) for dADT

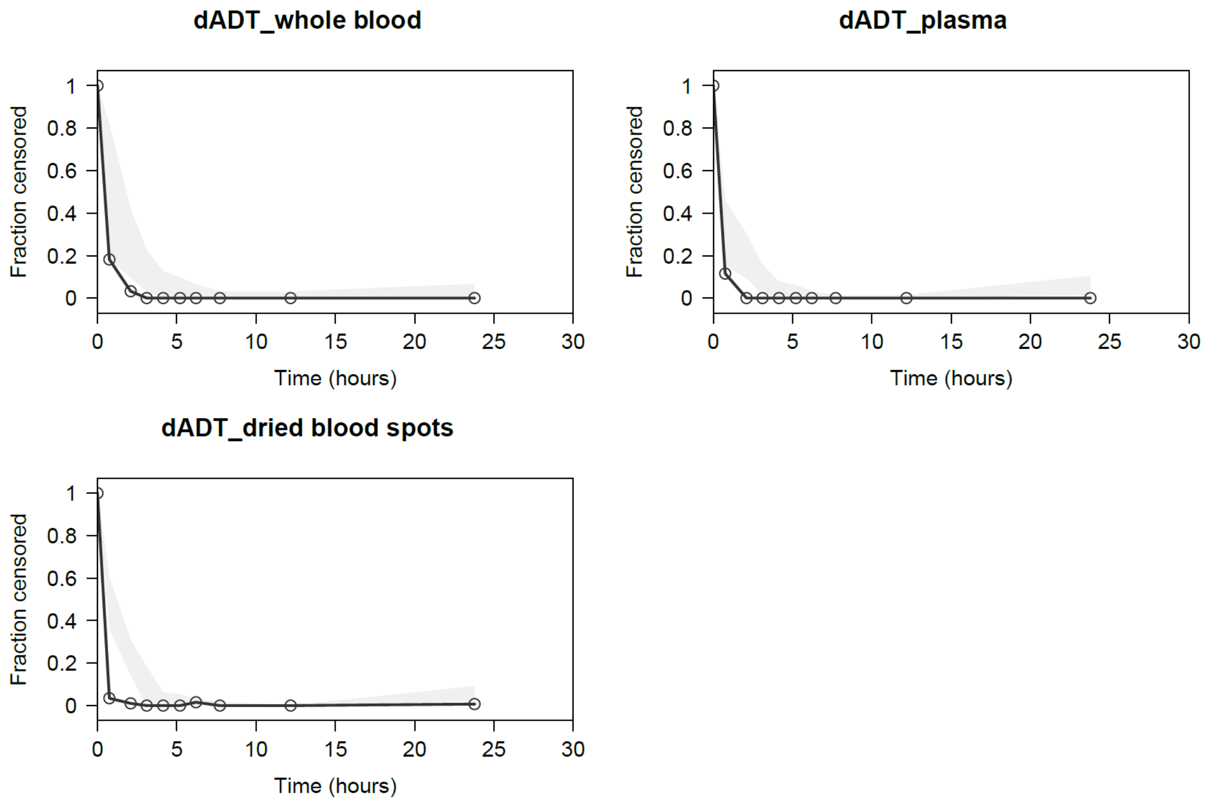

Figure S4 The fraction of observed data below the LLOQ (open circles) overlaid with the 95% prediction interval of the fraction of simulated data below the LLOQ (shaded area) for adADT

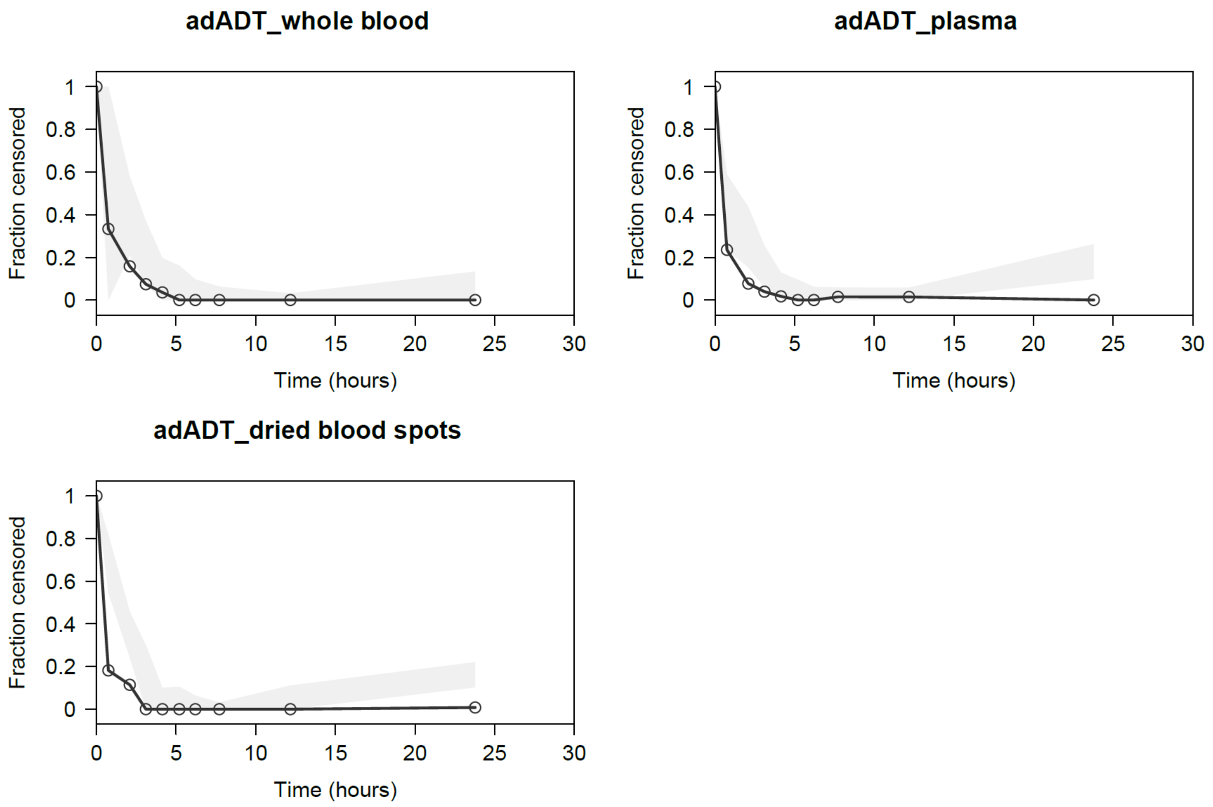

Figure S5 Goodness-of-fit diagnostics for dADT showing the observed versus population-predicted (A) and individually-predicted concentrations (B), conditional weighted residuals versus population predicted (C) and time (D) from the final population PK model.

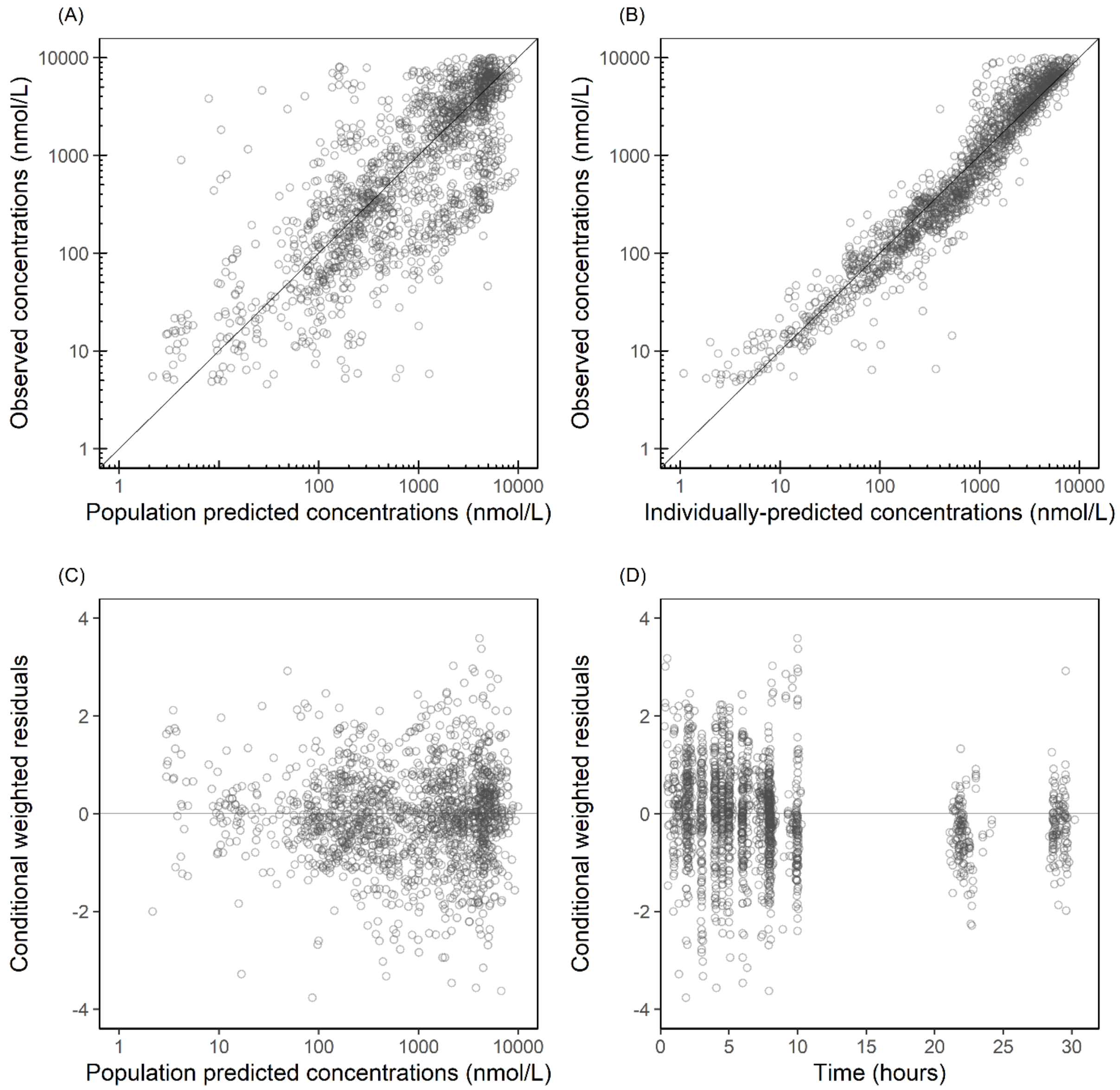

Figure S6 Goodness-of-fit diagnostics for adADT showing the observed versus population-predicted (A) and individually-predicted concentrations (B), conditional weighted residuals versus population predicted (C) and time (D) from the final population PK model.

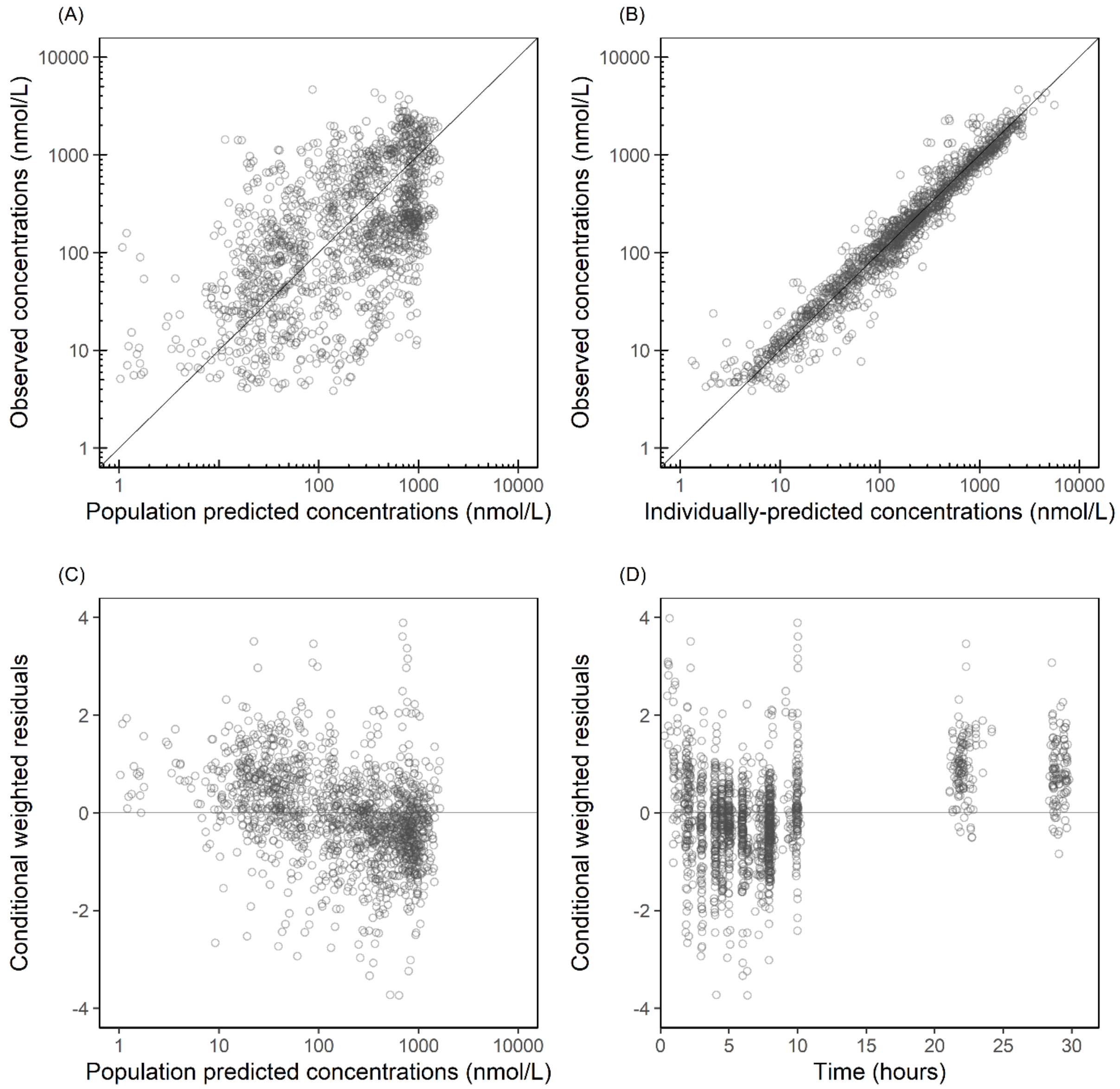

References

Duthaler, U., Sayasone, S., Vanobbergen, F., Penny, M., Odermatt, P., Huwyler, J. and Keiser, J. (2016) 'Single ascending dose pharmacokinetic study of tribendimidine in Opisthorchis viverrini-infected patients', *Antimicrobial Agents and Chemotherapy*, p. AAC. 00992-16.

Mentré, F., Duffull, S., Gueorguieva, I., Hooker, A., Leonov, S. and Ogungbenro, K. (2007) 'Software for optimal design in population PKPD: a comparison [Internet]. Population Approach Group in Europe '. Edited by A. from: http://www.page-meeting.org/pdf_assets/9481-mentre_page07postPage2.pdf.

Savic, R. M., Jonker, D. M., Kerbusch, T. and Karlsson, M. O. (2007) 'Implementation of a transit compartment model for describing drug absorption in pharmacokinetic studies', *Journal of Pharmacokinetics and Pharmacodynamics*, 34(5), pp. 711–726. doi: 10.1007/s10928-007-9066-0.

Xiao, S. H., Xue, J., Xu, L. L., Zheng, Q., Qiang, H. Q. and Zhang, Y. N. (2009) 'The in vitro and in vivo effect of tribendimidine and its metabolites against Clonorchis sinensis', *Parasitol Res*. 2009/08/06, 105(6), pp. 1497–1507. doi: 10.1007/s00436-009-1579-6.
